# Economic Evaluation of Pharmacopuncture for Adhesive Capsulitis Alongside a Pilot Pragmatic Randomized Controlled Trial Comparing Pharmacopuncture and Physical Therapy

**DOI:** 10.3390/healthcare14050605

**Published:** 2026-02-27

**Authors:** Doori Kim, Kyoung Sun Park, Sun-A Kim, Ji Yeon Seo, Hyun-Woo Cho, Yoon Jae Lee, Changsop Yang, Jung-Hee Jang, In-Hyuk Ha, Chang-Hyun Han

**Affiliations:** 1 Jaseng Hospital of Korean Medicine, 536 Gangnam-Daero, Gangnam-Gu, Seoul 06110, Republic of Korea; doori.k07@gmail.com (D.K.); lovepks0116@gmail.com (K.S.P.); 2 Daejeon Jaseng Hospital of Korean Medicine, 58, Munjeong-Ro 48Beon-Gil, Seo-Gu, Daejeon 35262, Republic of Korea; tnsdk2648@jaseng.org; 3 Bucheon Jaseng Hospital of Korean Medicine, 17, Buil-Ro 191Beon-Gil, Bucheon-Si 14598, Republic of Korea; wowpan21@jaseng.org; 4 Haeundae Jaseng Hospital of Korean Medicine, 100 Haeundae-Ro, Haeundae-Gu, Busan 48102, Republic of Korea; kamui0328@jaseng.org; 5 Jaseng Spine and Joint Research Institute, Jaseng Medical Foundation, 540 Gangnam-Daero, Gangnam-Gu, Seoul 06110, Republic of Korea; goodsmile8119@gmail.com; 6 KM Science Research Division, Korea Institute of Oriental Medicine, 217, Gajeong-Ro, Yuseong-Gu, Daejeon 34054, Republic of Korea; yangunja@kiom.re.kr (C.Y.); jee3838@kiom.re.kr (J.-H.J.); 7 Korean Convergence Medical Science, Korea Institute of Oriental Medicine School, University of Science & Technology, 1672, Yuseong-Daero, Yuseong-Gu, Daejeon 34054, Republic of Korea

**Keywords:** adhesive capsulitis, cost-effectiveness analysis, pharmacopuncture, physical therapy, pragmatic controlled trial, shoulder pain

## Abstract

**Background/Objectives**: Pharmacopuncture and physical therapy are commonly used to treat adhesive capsulitis (AC); however, their comparative cost-effectiveness is unclear. In this study, we aimed to investigate their cost-effectiveness for patients with AC. **Methods**: We conducted an economic evaluation alongside a 12-week, multicenter, pragmatic randomized controlled trial in four Korean medicine hospitals in South Korea. Patients with limited range of motion and pain score (numeric rating scale score ≥ 5) were randomized into a pharmacopuncture therapy or physical therapy group at a ratio of 1:1. Interventions were administered twice weekly for 6 weeks, with a follow-up of up to 12 weeks. Quality-adjusted life years were calculated using the EuroQol-5 Dimension 5 Level (EQ-5D-5L) and Short Form-6 dimension. Costs from societal and healthcare system perspectives were analyzed. **Results**: In total, 50 participants (pharmacopuncture therapy: 24; physical therapy: 26) were included. The differences in quality-adjusted life years between the groups were 0.014 and 0.013 when calculated using the EQ-5D-5L and Short Form-6-dimension scores, respectively. The costs from the societal perspective were significantly lower, whereas medical costs were higher, in the pharmacopuncture therapy group. Pharmacopuncture was the dominant treatment from the societal perspective. From the healthcare system perspective, the incremental cost-effectiveness ratios of pharmacopuncture to physical therapy were $4386 and $4790 when calculated using the EQ-5D-5L and Short Form-6-dimension scores, respectively. Sensitivity analyses confirmed the results. **Conclusions**: Pharmacopuncture therapy is more cost-effective for patients with AC than physical therapy. In this pilot study, pharmacopuncture may represent a potentially cost-effective treatment compared with physical therapy for patients with adhesive capsulitis, and the feasibility of conducting a full-scale study was confirmed.

## 1. Introduction

Shoulder pain is the third most common musculoskeletal symptom after lower back and knee pain [1]. It imposes a significant clinical and socioeconomic burden, with severe pain and substantial medical costs [2]. Although prevalence estimates vary, the 12-month prevalence of shoulder pain ranges from 11% to 55% [3]. In South Korea, the number of patients with shoulder pain increased from 1.4 million in 2008 to 2.4 million in 2018 [4]. Shoulder pain can interfere with work, social activities, and hobbies, causing psychological distress and affecting the quality of life [5].

Adhesive capsulitis (AC) or “frozen shoulder” is a common cause of shoulder pain [6]. AC is an inflammatory condition involving adhesions in the glenohumeral joint, resulting in pain, stiffness, functional loss, and limited range of motion (ROM) [7]. Affecting 2–5% of the population, AC is more common in women in their 40s to 60s [7,8]. It typically progresses through painful, stiff, and recovery phases, often requiring 1–2 years for symptom resolution [9]. However, persistent pain and functional disability due to AC are also possible [10,11].

Conservative treatments for AC management comprise oral medications, including non-steroidal anti-inflammatory drugs, oral corticosteroids, and analgesics [12,13], as well as non-pharmacological treatments, including intra-articular injections [14,15], physical or exercise therapy [16,17], and hydrodilation [18,19]. Nerve blocks [20,21] are used to treat severe symptoms. In clinical practice in Korea, different types of injections are administered to patients with AC, including prolotherapy, which involves the injection of concentrated glucose [22], polydeoxyribonucleotide [23], and injectable collagen [24]. In rare cases, surgical options (such as arthroscopic capsule release) are considered for patients with AC refractory to conservative treatment and with debilitating loss of ROM [25,26].

Complementary and alternative medicine is also sought for its efficacy and safety [27]. South Korea has a dual medical system for Korean medicine (KM) and Western medicine; many patients use the KM system for this purpose. Among the different treatment modalities available in KM, pharmacopuncture therapy (PPT), which combines the practices of acupuncture and herbal medicine in traditional KM, is one of the most commonly applied methods [28]. In PPT, herbal medicine extracts are injected into acupoints, leveraging both physical and chemical effects for enhanced treatment outcomes [29,30]. Survey-based studies show that PPT is frequently used to treat musculoskeletal disorders [31,32].

Nevertheless, high-quality evidence for the use of PPT in AC treatment is limited. A randomized controlled trial (RCT) reported the efficacy of bee venom acupuncture for AC [33], and a literature review showed that significant improvements in pain and functional disability were achieved in patients with AC through different types of PPT, including Ai-Tong-Shu, Danxiang injection, and Junfang-Danggui injection [34]. However, no previous studies have investigated the effectiveness of PPT as a treatment strategy for AC, and the findings of many existing studies are limited, owing to their small sample sizes, revealing limitations in the methodology. Therefore, we performed a pilot pragmatic RCT to examine the effectiveness of PPT in patients with AC and a cost–utility analysis to simultaneously evaluate its cost-effectiveness.

## 2. Materials and Methods

### 2.1. Study Design and Setting

In this 12-week pilot pragmatic RCT, patients with AC were recruited between April 2022 and September 2022. The results of a pragmatic RCT on PPT effectiveness and safety have been previously reported [35]. Briefly, 50 patients presenting with AC symptoms were randomized to the PPT or physical therapy (PT) group at a 1:1 ratio. Treatment sessions were conducted twice weekly for 6 weeks, and the participants were followed up for 12 weeks post-randomization.

### 2.2. Participants

This study included participants aged 19–69 years who had limitations in shoulder movement and intense shoulder pain (numeric rating scale score ≥ 5) persisting for more than a month. Detailed inclusion and exclusion criteria were consistent with those previously described [35].

### 2.3. Interventions

PPT was administered twice weekly for 6 weeks. The type of pharmacopuncture solution was selected based on the clinical judgment of KM doctors (KMDs), considering each patient’s condition. KMDs with >5 years of clinical experience performed the intervention. Intervention details (e.g., the pharmacopuncture solution used and acupoints selected) were recorded in the electronic medical record (EMR) and case report form (CRF).

In the control group, PT was administered twice weekly for 6 weeks. The PT method, treatment area, and duration were determined by the physician based on symptoms, radiological findings, and improvement. All PT details, including type, frequency, and application area, were recorded in the EMR and CRF.

### 2.4. Utilities

The EuroQol-5 Dimension 5 Level (EQ-5D-5L) and Short Form-12 scores were measured at baseline and at 8 and 12 weeks post-randomization. The validated Korean version of the EQ-5D-5L was used, and utility scores were calculated using the Korea-specific tariff by Kim et al. [36]. Health-related quality of life was assessed using the 12-Item Short Form Health Survey version 2 (SF-12v2), and the scores were converted to Short Form-6 Dimension (SF-6D) values using the equation by Brazier et al. [37] for cost–utility analysis. Quality-adjusted life years (QALYs) were calculated using the area under the curve and trapezoidal rules, adjusted for baseline utility values [38].

### 2.5. Unit Costs

Data sources for the cost of interventions administered during the trial along with the unit costs are presented in Table 1. As PPT is not reimbursed by the National Health Insurance system, its price may vary depending on the KM hospitals/clinics. Therefore, we based the unit cost on empirical pricing patterns and nationally reported statistics from the Korean Statistical Information Service (KOSIS). According to KOSIS, the median cost of pharmacopuncture was KRW 10,000 in Korean medicine clinics and KRW 30,000 in Korean medicine hospitals [39]. In routine musculoskeletal practice, most pharmacopuncture procedures fall within this range. To avoid overestimation and reflect real-world distribution, we conservatively set the base-case unit cost at KRW 20,000 (USD 17), representing the midpoint between clinic and hospital medians. Information on the consultation fee and charges for PT was extracted from the 2022 Health Insurance Medical Care Benefit Expenses from the Health Insurance Review & Assessment Service, and costs were calculated based on treatment type and frequency.

### 2.6. Resource Use Measurements

Information on healthcare services used for AC treatment (excluding the trial interventions) was collected during each visit via patient questionnaires. Items included costs of non-reimbursed services, patient co-payments, and frequency of additional service use. Payer reimbursement was calculated by analyzing data from the 2019 Health Insurance Review and Assessment Service–National Patient Sample according to sex and age [40].

Transportation costs were calculated from the patient survey. The time patients spent receiving treatment for AC was surveyed. Based on this information, the time cost was calculated by substituting the standard wages for individual patients according to sex and age provided in the 2022 Survey Report on Labor Conditions by Employment Type [41].

Productivity loss was measured using the Work Productivity and Activity Impairment–Specific Health Problem (WPAI-SHP) questionnaire [42] at baseline and at 2, 3, 4, 5, 8, and 12 weeks post-randomization. The WPAI-SHP assessed absenteeism, presenteeism, overall productivity loss, and activity impairment over the previous 7 days owing to AC [43,44]. We included productivity loss from paid employment, self-employment, and household work. WPAI scores were calculated by applying overall work productivity loss to employed participants and activity impairment to others. Economic losses were estimated by multiplying WPAI values by the standard wage according to sex and age [41]. A discount rate was not applied due to the 12-week study period. All costs were converted using the 2022 exchange rate (1264.5 KRW = $1).

### 2.7. Economic Viewpoint

For the economic evaluation, a primary analysis was conducted from the societal perspective. Societal costs included direct medical costs, direct non-medical costs, and costs of productivity loss. Direct medical costs included the cost of interventions applied during this trial and costs incurred during the study period, including the follow-up period (e.g., the cost of treatment for AC from other medical institutions, cost of analgesics including over-the-counter drugs, and cost of informal care, including medical devices or exercise therapy). Direct non-medical costs included intervention time and transportation costs. Economic evaluation was performed from the healthcare system perspective in addition to the societal perspective, and only direct medical costs were considered for this analysis.

### 2.8. Data Analysis

The primary aim of this study was to evaluate the incremental cost utility from a societal perspective. Here, the differential mean costs and QALYs were compared between the PPT and PT groups using the independent t-test. The incremental cost-effectiveness ratio (ICER) was calculated by dividing the difference in total costs by that in QALYs.

Intention-to-treat (ITT) analysis was used to analyze the trial results. In the pharmacopuncture group, one participant dropped out at week 7 (end of intervention), and one additional participant dropped out by week 12 (final follow-up), resulting in two dropouts in total. Accordingly, missing data rates for key variables, including SF-12, EQ-5D-5L utilities, WPAI, and medical costs, were 2% (1/50) at week 7 and 4% (2/50) at week 12. No additional missing values were observed among participants who completed follow-up. Missing values were imputed with multiple imputations using the Markov Chain Monte Carlo method and predictive mean matching. Twenty imputed datasets were generated, and the covariates for imputation were treatment allocation, sex, age, and body mass index. The “mice” package in R software (version 4.0.1; R Core Team, Vienna, Austria) was used to impute the missing values.

### 2.9. Uncertainty

The uncertainty of the ICER was estimated using the bootstrap residual technique [38]. To enable valid bootstrap inference with multiple imputations (MIs), 10,000 datasets were generated by applying 500 replications to 20 MI sets, according to the methodology proposed by Schomaker et al. [45] in which a bootstrap is recommended for each MI set. First, estimates were derived from the linear regression on 20 MI sets and then used to calculate predicted outcomes and residuals. Second, regression residuals were bootstrapped, and the group difference was calculated by adding bootstrapped residuals to predicted outcomes [46]. The uncertainty of the ICER was visualized as incremental cost–effect pairs on cost-effectiveness planes (CE planes), with the proportion of pairs distributed in each quadrant derived. Cost-effectiveness acceptability curves were also generated to show the probability of PPT being cost-effective according to willingness to pay (WTP). A WTP threshold of KRW 30.5 million ($26,374) per QALY was used, based on empirically elicited societal WTP values and commonly adopted as a practical benchmark [47]. Multiple Korean CEAs explicitly apply KRW 30–20.5 million/QALY as the reference threshold [48,49,50]. In addition, it is also broadly comparable to an approximately 1 GDP-per capita benchmark in Korea in many recent years [51,52]. In the probabilistic sensitivity analysis, we evaluated how the probability of pharmacopuncture being cost-effective changed across varying WTP thresholds. Sensitivity analyses included the following: (1) per-protocol (PP) analysis for patients receiving ≥ 9 treatments; (2) analysis from a healthcare system perspective including medical and non-medical costs; (3) cost–utility analysis assuming that pharmacopuncture fees were 1.5× and 2× those in the base case, reflecting institutional variation; (4) alternative productivity loss estimation (whereas the base case applied impairment to all participants, this scenario assumed that productivity loss occurred only among employed patients); and (5) economic evaluation assuming that the values of EQ-5D-5L, SF-6D, healthcare use costs, and productivity loss at week 12 were sustained for 1 year.

### 2.10. Ethics and Protocol Registration

The study protocol complied with the Consolidated Standards of Reporting Trials guidelines. The protocol is registered with ClinicalTrials.gov (NCT05292482) and cris.nih.go.kr (KCT0007198). The study was approved by the institutional review board of Jaseng Hospital of Korean Medicine prior to the recruitment of patients (approval numbers: JASENG 2022-02-013, JASENG 2022-02-014, JASENG 2022-02-015, and JASENG 2022-02-016, approved date: 11 March 2022). This study complied with the principles of the Helsinki Declaration. Before participation, the investigators fully explained the study-related information (effects, adverse events [AEs], and safety) to each participant individually and obtained written informed consent.

## 3. Results

### 3.1. Participant Characteristics and Treatment Details

**Figure 1:**
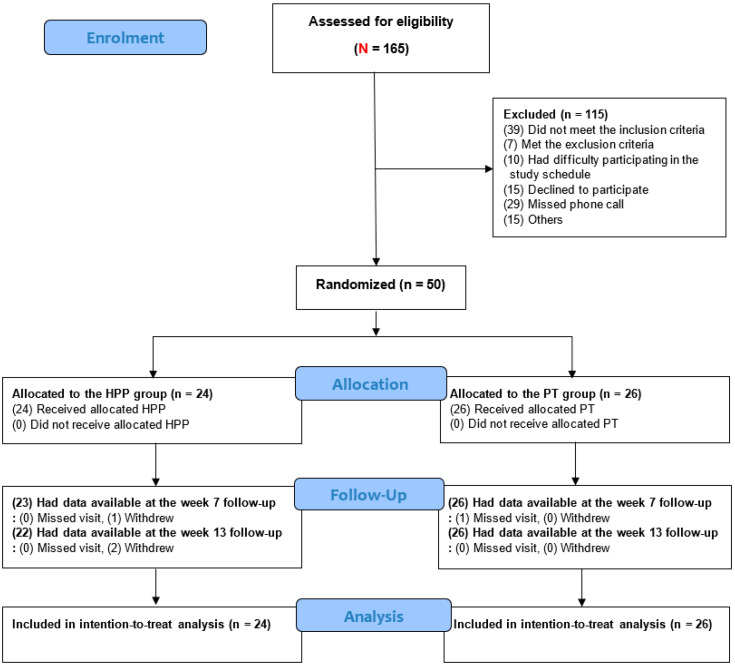
Flowchart of participant selection. Two participants in the pharmacopuncture group withdrew consent (one at week 7 and one at week 12). No additional reasons were documented.

During the study period, 165 patients underwent screening; of these, 50 met the eligibility criteria and were enrolled (Figure 1). By randomization, 24 and 26 patients were assigned to the PPT and PT groups, respectively; all participants were included in the ITT analysis. The baseline characteristics of the participants are summarized in Tables S1–S3. There were no significant differences in baseline characteristics between the groups.

Analysis of treatment details revealed that Shinbaro 2 pharmacopuncture was the most frequently used formulation in the PPT group, and Jianjing (GB21) and Jianyu (LI15) were the most commonly selected acupoints. In the PT group, interferential current therapy (ICT) and deep heat therapy were the most frequently administered modalities (Table S4).

### 3.2. QALYs

**Figure 2:**
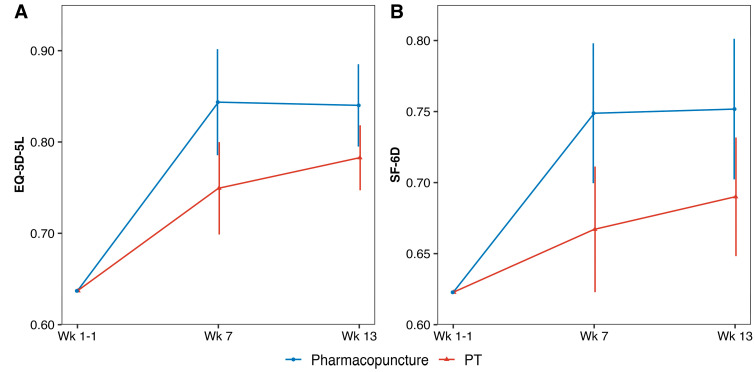
Distribution of EQ-5D-5L and SF-6D scores by group. EQ-5D-5L, EuroQol-5 Dimension 5-Level; PT, Physical therapy; SF-6D, Short Form-6 Dimension.

The QALYs are shown in Table 2 and Figure 2. The adjusted QALYs of the PPT and PT groups after 12 weeks were 0.183 and 0.168, respectively. The difference in QALYs calculated based on the EQ-5D score was 0.014 (95% confidence interval [CI]: 0.003–0.025), and the QALYs of the PPT group were significantly higher than those of the PT group (*p* = 0.013). The adjusted QALYs calculated based on the SF-6D score were 0.166 and 0.153 for the PPT and PT groups, respectively. The difference in QALYs was 0.013 (95% CI: 0.003–0.023), indicating that the QALYs of the PPT group were significantly higher than those of the PT group (*p* = 0.011).

### 3.3. Costs

The medical costs at each time-point are presented in Table 3. The medical costs were $301 (95% CI: $271–321) in the PPT group and $239 (95% CI: $213–280) in the PT group; the cost was significantly higher in the PPT group than in the PT group (difference: $62, 95% CI: $14–97, *p* = 0.016). In both groups, almost no cost was incurred from using additional health services during the follow-up period, and most of the medical costs corresponded to the interventions performed during the intervention period. The non-healthcare cost, including transportation and time, was higher in the PT group than in the PPT group (difference: −$63, 95% CI: −$188 to $71). When examining the time cost in more detail, the waiting time was longer in the PPT group than in the PT group, whereas the consultation and treatment times were shorter in the PPT group than in the PT group (Tables S1–S3). The costs of productivity loss were $2235 (95% CI: $1665–2841) and $3759 (95% CI: $3200–4377) in the PPT and PT groups, respectively, showing a significantly lower cost in the PPT group than in the PT group (*p* = 0.002). Therefore, the total cost from the societal perspective, which included medical, non-medical, and productivity loss costs, was significantly lower in the PPT group than in the PT group (difference: -$1525, 95% CI: −$2379 to −$698, *p* = 0.002). Additional cost details are presented in Tables S1–S3.

### 3.4. Cost–Utility Analysis

**Figure 3:**
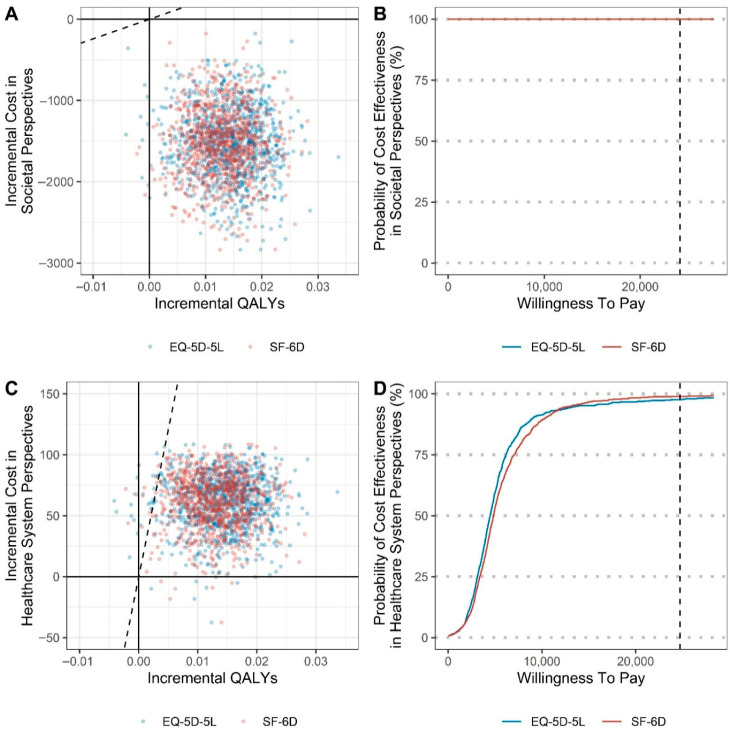
Comparison of the cost-effectiveness plane and cost-effectiveness acceptability curves for pharmacopuncture and physical therapy. EQ-5D-5L, EuroQol-5 Dimension 5-Level; QALYs, Quality-adjusted life years; SF-6D, Short Form-6 Dimension.

The QALYs were significantly higher in the PPT group than in the PT group, and the cost from the societal perspective was lower in the PPT group than in the PT group. PPT was the dominant treatment method compared to PT. Based on the WTP determined through a survey of the general population of South Korea, which was $26,375, the probability that PPT was cost-effective from the societal perspective was 100%. From the healthcare perspective, the ICERs were $4386 and $4790 when calculated using the EQ-5D-5L and SF-6D scores, respectively, and the probability that PPT was cost-effective was >97% according to the WTP (Table 4, Figure 3).

### 3.5. Sensitivity Analysis

The results of the different sensitivity analyses are presented in Table 4. First, a cost-effectiveness analysis was performed only in patients who received more than nine treatments (PP analysis). The differences in costs between the two groups were −$1508 from the societal perspective and $70 from the healthcare perspective. From the societal perspective, PPT was the dominant treatment, and the probability of PPT being cost-effective was 100% at 1 WTP. From the healthcare perspective, the ICERs were $4506 and $5105 when calculated using the EQ5D and SF-6D scores, respectively, and the probability of PPT being cost-effective was >98% at 1 WTP.

Second, an analysis was conducted from the healthcare system perspective, including medical and non-medical costs. The difference in cost between the two groups was −$1, and PPT was the dominant treatment. The probability of PPT being cost-effective was also >98% at 1 WTP.

Third, the pharmacopuncture fee was 1.5 and 2 times that in the primary analysis. Even with such increases in the fees for pharmacopuncture services, PPT remained the dominant treatment from a societal perspective, and the probability of PPT being cost-effective was 100% at 1 WTP. As the cost difference from the healthcare system perspective increased, the ICER values were as large as $10,655 and $16,925 when calculated using the EQ-5D score, and $11,637 and $18,485 when calculated using the SF-6D score, respectively.

Fourth, a cost-effectiveness analysis was performed by altering the method for calculating productivity loss costs to include only the impairment in overall work for those engaged in paid employment. The difference in the cost from the societal perspective was $1539, which did not show a notable difference from the −$1525 in the main analysis.

Finally, a sensitivity analysis was conducted, assuming that the values at week 12 were maintained for 1 year. The cost difference from the societal perspective was $6885, whereas that from the healthcare system perspective was $81. The differences in the QALYs calculated using the EQ-5D-5L and SF-6D scores were 0.058 and 0.060, respectively. Therefore, PPT remained the dominant treatment from the societal perspective. The ICERs from the healthcare system perspective were $1396 when calculated using the EQ-5D-5L score and $1346 when calculated using the SF-6D score.

## 4. Discussion

In this study, an economic evaluation was conducted alongside a pilot RCT comparing the effectiveness of PPT and PT in patients with AC. This pilot pragmatic RCT [35] showed that PPT was more effective than PT in reducing pain, improving function, and increasing ROM. It also confirmed the feasibility of a full-scale study. In this accompanying economic evaluation, PPT was a dominant strategy, showing higher QALYs and lower societal costs than PT. Although the healthcare system cost was higher for PPT, the ICER remained below $5000. The probability of PPT being cost-effective at 1 WTP exceeded 95%, supporting its cost-effectiveness.

The difference in QALYs between the two groups calculated using the EQ-5D-5L and SF-6D scores was significant. It was also significant in the PP analysis and further increased under the assumption that week 12 values were sustained for 1 year. This was mainly driven by significant differences in EQ-5D-5L and physical component summary (PCS) scores. This is interesting considering that in many clinical trials of patients without life-threatening musculoskeletal pain (e.g., neck and back pain), no significant difference in the quality of life was found between the groups [53,54]. It can be inferred that AC affects the quality of life of patients more than other musculoskeletal disorders.

Quality of life reflects physical, psychological, and social aspects [55]. Considering that the EQ-5D-5L emphasizes physical functioning [56] and that significant group differences were observed in PCS but not in the mental component summary score, the findings suggest that the compromised quality of life in AC is closely linked to limitations in physical function such as stiffness and restricted ROM. Therefore, PPT may be a cost-effective option for patients whose quality of life is reduced by physical limitations due to AC.

Owing to the higher fee for PPT than for PT, the cost from the healthcare system perspective was higher in the PPT group than in the PT group. However, the calculated ICER was low because of the considerable differences in QALY values between the groups. Moreover, most of the bootstrapped points in the CE planes were located below the ICER slope, and in the cost-effectiveness acceptability curves, the probability that PPT was cost-effective reached 100% with the cost approaching 1 WTP. When the fee for the pharmacopuncture service was increased by 1.5 or 2 times in relation to that considered for the primary analysis, the medical cost difference between the groups was further increased. However, the ICER value was not large but acceptable, because the difference in QALYs between the groups was substantial, and the probability of PPT being cost-effective at 1 WTP was >70%. In this study, productivity losses were estimated using the human capital approach (HCA), which values lost work time based on forgone earnings, while the friction cost approach (FCA) limits productivity loss to the replacement period. Although both approaches are debated, the HCA remains widely used in economic evaluations and is recommended in many guidelines. [57] Therefore, we adopted the HCA in the base-case analysis.

A substantial proportion of participants (28 of 50) were not in paid employment. We therefore included absenteeism and presenteeism among employed participants and considered activity impairment for unemployed individuals (e.g., homemakers and self-employed persons) in the base-case analysis [58]. The overall work impairment score, calculated for all participants, showed significant differences at week 7 (end of intervention) and week 12 (follow-up) (Tables S1–S3).

Although productivity loss differences were the main driver of societal cost differences, sensitivity analysis restricting productivity loss calculations to employed participants only still demonstrated pharmacopuncture to be dominant over physical therapy. Moreover, under the healthcare system perspective—excluding productivity costs—pharmacopuncture remained cost-effective, with ICER values well below the predefined WTP threshold.

While the use of HCA and the inclusion of activity impairment among unemployed participants may potentially overestimate productivity-related differences, multiple sensitivity analyses confirmed the robustness of the conclusion that pharmacopuncture is likely to be cost-effective compared with physical therapy.

When non-medical costs were analyzed in more detail, the time costs for consultation and treatment were lower in the PPT group than in the PT group. This is because PPT involves using a syringe to administer pharmacopuncture solution at therapeutic points (acupoints), which requires a shorter time than other types of treatment such as PT. These results confirmed that PPT is a highly effective treatment method that requires only a short time.

Notably, the PT group also showed improvement in efficacy outcomes above the level of the minimal clinically important difference. Moreover, the quality of life outcomes improved after PT. PT is a treatment method recommended for AC [59], and several RCTs have reported that PT improves pain scores, functional scores, and ROM [60]. Therefore, PT is often used alone or in combination with other treatments in the “usual care” group when evaluating the effectiveness of a specific treatment method [61]. Here, the treatment program for the PT group was developed by combining the most frequently used PT methods in Korea [6], with the findings indicating that PPT has superior cost-effectiveness to PT. This has relevant implications for patients with AC.

This study has limitations. First, as an open-label trial, it was susceptible to bias due to lack of blinding; however, assessor blinding was implemented to mitigate this.

Second, as a pragmatic trial, interventions were delivered with clinician discretion to reflect routine practice, which inevitably introduces within-group heterogeneity. Nevertheless, to maintain interpretability, we predefined core treatment principles and a fixed treatment schedule while systematically documenting key treatment components. The primary analysis used intention-to-treat principles with baseline adjustment and repeated-measure modeling, and randomization mitigates bias by balancing measured and unmeasured factors across groups. Therefore, our estimates represent the average treatment effect under real-world practice conditions.

Third, this economic evaluation was conducted in the Korean decision context; therefore, several inputs are jurisdiction-specific, including unit costs based on HIRA wage data used to value time and productivity losses, the Korean EQ-5D-5L tariff, and the willingness-to-pay threshold. Cost-effectiveness results are generally not directly transferable across jurisdictions without adaptation of these context-dependent parameters [62]. Nevertheless, the clinical effectiveness estimates and within-trial resource-use patterns observed in this pragmatic trial may be informative for other settings with comparable care pathways. To facilitate adaptation, we reported cost components in a disaggregated manner so that analysts in other jurisdictions can substitute local unit costs, utility tariffs, and decision thresholds.

A further limitation is that minimum and maximum national price data specific to musculoskeletal pharmacopuncture were not available. Consequently, sensitivity analyses were conducted using 1.5× and 2× multipliers of the base-case unit cost rather than empirically derived minimum and maximum values. Although the base-case cost was conservatively defined, the absence of formally documented price extremes should be acknowledged.

Lastly, interpretation requires caution, as the economic evaluation was based on a pilot study with a relatively small sample size (n = 50) and a short follow-up period of 12 weeks. Given the typically prolonged clinical course of adhesive capsulitis, often lasting 1–2 years, the limited time horizon represents an important limitation. Nevertheless, as a pilot study, the primary objective was to assess feasibility and generate preliminary economic data to inform the design of a full-scale trial. Future adequately powered randomized controlled trials with longer follow-up periods are needed to confirm the long-term cost-effectiveness of pharmacopuncture.

Despite the limitations, the study represents a robust economic evaluation alongside a pragmatic RCT, which is well suited for real-world cost-effectiveness analysis [63]. To our knowledge, no prior study has evaluated the cost-effectiveness of PPT in AC.

## 5. Conclusions

In this pilot study, it was found that pharmacopuncture may represent a potentially cost-effective treatment compared with physical therapy for patients with adhesive capsulitis, and the feasibility of a larger trial was demonstrated. A full-scale randomized controlled trial with a larger sample size and longer follow-up is warranted to confirm these findings.

## Figures and Tables

**Table 1:** Cost calculation method, associated data source, and unit cost.

Type of Cost	Calculation Method	Data Source	Unit Cost ($)
Pharmacopuncture	Pharmacopuncture is a noninsurance-covered treatment. Costs commonly prescribed by medical institutions were investigated and applied.		16
Physical therapy	The applied costs corresponded to the HIRA price index.	HIRA price index 2022	7 [4, 67]
Consultation fee at the first visit with the Korean medicine doctor	When a patient visits a medical institution, a medical doctor provides a diagnosis. The consultation fee is set differently for the first and recursive visits. Additionally, the consultation fee is billed differently depending on the type of physicians (Korean medicine doctor or general practitioner). The pharmacopuncture therapy group was consulted by a Korean medicine doctor and the physical therapy group by a general practitioner.	HIRA price index 2022	12
Consultation fee at recursive visits with the Korean medicine doctor		HIRA price index 2022	8
Consultation fee at the first visit with the Western medicine doctor		HIRA price index 2022	15
Consultation fee at recursive visits with the Western medicine doctor		HIRA price index 2022	11
Syndrome differentiation technique fee	Korean traditional medicine has a system that examines comprehensive symptoms based on unique theories and experiences, i.e., syndrome differentiation (辨證). All Korean medicine doctors may charge an additional syndrome differentiation technique fee (辨證技術料) according to the examination. This can be charged once a week, and the patients visited up to twice a week during the 5-week intervention period.	HIRA price index 2022	3
Additional private outpatient visits	The patient’s visits to medical institutions other than clinical trial sites and the associated expenses were surveyed. In this case, the amount indicated in the patient’s response was out-of-pocket payments for health insurance and non-covered services. Thus, the benefits (i.e., insurance-covered cost) were unknown. Accordingly, the benefits were calculated from the claim data of temporomandibular disorder in the HIRA-NPS from 2019. The calculated average benefits were stratified by the patient’s sex and age and matched.	Patient survey and HIRA-NPS 2019	36 [21, 71]
Transportation	The transportation cost to visit the clinical trial site was surveyed 1 week after the baseline, and this was multiplied by the number of visits.	Patient survey	3 [2, 9]
Time costs for the intervention	After 1 week from the baseline, the total time taken for the patient to leave the house, travel to the hospital, interview, wait, receive treatment, and return home was recorded and then multiplied by the number of visits.	Patient survey and income	33 [13, 109]
Productivity cost	Productivity loss was assessed using WPAI-SHP. Then, according to the human capital approach, productivity loss was multiplied by sex- and age-stratified income to calculate the income loss due to the productivity loss, which is regarded as the productivity cost. In the base case analysis, overall work impairment was applied to the productivity loss of employed patients, and activity impairment was applied to unemployed patients. The result of estimating the productivity costs only for employed patients is presented in the sensitivity analysis.	Patient survey and 2022 Survey Report on Labor Conditions by Employment Type	—

HIRA, Health Insurance Review & Assessment Service; HIRA-NPS, Health Insurance Review & Assessment Service–National Patient Sample; WPAI-SHP, Work Productivity and Activity Impairment–Specific Health Problem.

**Table 2:** Utility and quality-adjusted life years after randomization by group.

	PPT Group (n = 24)	PT Group (n = 26)	Difference	*p*-Value
EQ-5D				
Week 7	0.84 (0.79–0.90)	0.75 (0.70–0.80)	0.09 (0.02–0.17)	0.016
Week 13	0.84 (0.80–0.88)	0.78 (0.75–0.82)	0.06 (0.00–0.11)	0.048
QALY	0.183 (0.174–0.191)	0.168 (0.161–0.176)	0.014 (0.003–0.025)	0.013
SF-6D				
Week 7	0.75 (0.70–0.80)	0.67 (0.62–0.71)	0.08 (0.02–0.15)	0.015
Week 13	0.75 (0.70–0.80)	0.69 (0.65–0.73)	0.06 (0.00–0.13)	0.058
QALYs	0.166 (0.158–0.173)	0.153 (0.146–0.159)	0.013 (0.003–0.023)	0.011

EQ-5D, EuroQol-5 Dimension; PT, Physical therapy; PPT, Pharmacopuncture therapy; QALYs, Quality-adjusted life years; SF-6D, Short Form-6 Dimension.

**Table 3:** Comparison of the cost per patient between the study groups.

	PPT Group ($, 95% CI)	PT Group ($, 95% CI)	Difference ($, 95% CI)	*p*-Value
**Medical cost**				
Intervention	296 (270–311)	236 (210–274)	59 (13–94)	0.022
Follow-up	6 (0–13)	3 (0–8)	3 (−5–13)	0.627
Total	301 (271–321)	239 (213–280)	62 (14–97)	0.016
**Non-medical cost**				
Total	371 (267–484)	434 (362–507)	−63 (−188–71)	0.344
**Productivity loss**				
Intervention	1263 (985–1570)	1981 (1728–2227)	−(−1082 to −336)	0.004
Follow-up	972 (637–1321)	1779 (1423–2133)	−807 (−1288 to −313)	0.002
Total	2235 (1665–2841)	3759 (3200–4377)	−1524 (−2382 to −704)	0.002
**Societal perspective**				
Intervention	1930 (1626–2222)	2651 (2396–2942)	−722 (−1129 to −330)	0.002
Follow-up	977 (642–1341)	1781 (1424–2177)	−804 (−1312 to −286)	0.002
Total	2907 (2352–3454)	4432 (3810–5021)	−1525 (−379 to −698)	0.002

The study period was 12 weeks, and the “intervention” (period) refers to the 6 weeks during which the interventions were administered. Between-group differences were analyzed using the independent t-test. PT, Physical therapy; PPT, Pharmacopuncture therapy.

**Table 4:** Results of the cost-effectiveness analysis of pharmacopuncture therapy compared to physical therapy.

Analysis	Cost	Difference in Cost ($, 95% CI)	QALY Measure	Difference in QALYs	ICER ($)	Probability of the Cost-Effectiveness at 1 × WTP
Main analysis	Societal	−1525 (−2379 to −698)	EQ-5D-5L	0.014 (0.003–0.025)	Dominant	100
			SF-6D	0.013 (0.003–0.023)	Dominant	100
	Healthcare	62 (14–97)	EQ-5D-5L	0.014 (0.003–0.025)	4386	97.6
			SF-6D	0.013 (0.003–0.023)	4790	98.9
Sensitivity analysis 1 *	Societal	−1508 (−2357 to −638)	EQ-5D-5L	0.015 (0.005–0.026)	Dominant	100
			SF-6D	0.014 (0.004–0.023)	Dominant	100
	Healthcare	70 (27–98)	EQ-5D-5L	0.015 (0.005–0.026)	4506	99.7
			SF-6D	0.014 (0.004–0.023)	5105	98.8
Sensitivity analysis 2 ^†^	Healthcare + non-healthcare	−1 (−142–154)	EQ-5D-5L	0.014 (0.003–0.025)	Dominant	98.5
			SF-6D	0.013 (0.003–0.023)	Dominant	99.1
Sensitivity analysis 3-1 ^‡^	Societal	−1436 (−2303 to −598)	EQ-5D-5L	0.014 (0.003–0.025)	Dominant	100
			SF-6D	0.013 (0.003–0.023)	Dominant	99.9
	Healthcare	151 (102–190)	EQ-5D-5L	0.014 (0.003–0.025)	10,655	92.4
			SF-6D	0.013 (0.003–0.023)	11,637	92
Sensitivity analysis 3-2 ^§^	Societal	−1348 (−2163 to −493)	EQ-5D-5L	0.014 (0.003–0.025)	Dominant	100
			SF-6D	0.013 (0.003–0.023)	Dominant	100
	Healthcare	240 (184–284)	EQ-5D-5L	0.014 (0.003–0.025)	16,925	79.7
			SF-6D	0.013 (0.003–0.023)	18,485	72.8
Sensitivity analysis 4 ^| |^	Societal	−1539 (−2824 to −217)	EQ-5D-5L	0.014 (0.003–0.025)	Dominant	99.9
			SF-6D	0.013 (0.003–0.023)	Dominant	99.7
Sensitivity analysis 5 ^¶^	Societal	−6885 (−11,156 to −2537)	EQ-5D-5L	0.058 (0.006–0.111)	Dominant	100
			SF-6D	0.060 (0.003–0.117)	Dominant	100
	Healthcare	81 (−1 to 160)	EQ-5D-5L	0.058 (0.006–0.111)	1396	98.7
			SF-6D	0.060 (0.003–0.117)	1346	97.5

* Per-protocol analysis was performed for patients who received fewer than nine treatment sessions. ^†^ Costs from the healthcare system perspective were calculated, including medical and non-medical costs. ^‡^ The fee for the pharmacopuncture service was set to 1.5 times that in the main analysis to calculate the cost. ^§^ The fee for the pharmacopuncture service was set to 2 times that in the main analysis to calculate the cost. ^| |^ The productivity loss cost was calculated only for patients engaged in paid employment. ^¶^ The calculation assumed that the values of medical costs and quality-adjusted life years of life measures at week 12 were maintained for 12 months. EQ-5D-5L, EuroQol-5 Dimension 5-Level; ICER, Incremental cost-effectiveness ratio; PT, Physical therapy; PPT, Pharmacopuncture therapy; QALYs, Quality-adjusted life years; SF-6D, Short Form-6 Dimension; WTP, Willingness to pay.

## Data Availability

All data in this study were extracted from publicly available academic literature. Data presented are available from the corresponding author upon reasonable request.

## Supplementary Materials

The following supporting information can be downloaded at: https://www.mdpi.com/article/10.3390/healthcare14050605/s1. Table S1: Baseline characteristics of the participants; Table S2: Cost calculation by group; Table S3: Overall work impairment according to the Work Productivity and Activity Impairment score; Checklist S1: CONSORT 2010 checklist. Ref. [64] is cited in Supplementary Materials.

## Abbreviations

The following abbreviations are used in this manuscript:

AC	adhesive capsulitis
CE	cost-effectiveness
CI	confidence interval
CRF	case report form
EMR	electronic medical record
EQ-5D-5L	EuroQol-5 Dimension 5 Level
ICER	incremental cost-effectiveness ratio
ITT	intention-to-treat
KM	Korean medicine
KMD	Korean medicine doctor
MI	multiple imputation
PCS	physical component summary
PP	per-protocol
PPT	pharmacopuncture therapy
PT	physical therapy
QALY	quality-adjusted life year
RCT	randomized controlled trial
ROM	range of motion
SF-12v2	12-Item Short Form Health Survey version 2
SF-6D	Short Form-6 Dimension
WPAI-SHP	Work Productivity and Activity Impairment–Specific Health Problem
WTP	willingness to pay
