# Supplementary material for: Economic Evaluation of Pharmacopuncture for Adhesive Capsulitis Alongside a Pilot Pragmatic Randomized Controlled Trial Comparing Pharmacopuncture and Physical Therapy
Source: Healthcare (Basel). 2026 Feb 27;14(5):605. doi: 10.3390/healthcare14050605 (PMC12985154; doi:10.3390/healthcare14050605)
Supplement: Supplementary file 1 [file healthcare-14-00605-s001.zip › Tables S1-S4.pdf]

Supplementary Tables

Table S1. Baseline characteristics of the participants

		PPT group (n = 24)	PT group (n = 26)	p-value[a]
Sex				
	Female	14 (58.3)	14 (53.8)	0.973
	Male	10 (41.7)	12 (46.2)	
Age (years)		53.9 (8.6)	53.7 (7.5)	0.923
Height (cm)		164.7 (6.9)	166.0 (6.8)	0.503
Weight (kg)		65.3 (13.3)	66.4 (14.4)	0.785
BMI (kg/m^2)		24.0 (4.1)	23.9 (3.8)	0.953
Hypertension (%)		7 (29.2)	5 (19.2)	0.624
Hyperlipidemia (%)		3 (12.5)	2 (7.7)	0.6613
Diabetes mellitus (%)		1 (4.2)	1 (3.8)	1
Site of pain				
	Left	7 (29.2)	6 (23.1)	0.867
	Right	17 (70.8)	20 (76.9)	
Months from symptom onset		22.1 (52.9)	21.8 (25.3)	0.981
Degeneration		3 (12.5)	7 (26.9)	0.2938
Calcification		4 (16.7)	1 (3.8)	0.1815
Osteopenia		2 (8.3)	1 (3.8)	0.602
Prior medications		3 (12.5)	5 (19.2)	0.7041
Expectations of treatment scale,pharmacotherapy score		7.4 (1.1)	7.5 (1.1)	0.688
Expectations of treatment scale,usual care score		5.6 (1.4)	5.8 (1.4)	0.581
NRS score		6.8 (1.0)	6.7 (0.8)	0.941
VAS score		69.0 (10.3)	68.1 (9.0)	0.737
SPADI				
	Pain	68.7 (12.6)	72.7 (9.8)	0.213
	Function	58.8 (16.1)	64.8 (12.7)	0.148
	Total	62.6 (14.0)	67.8 (11.0)	0.145
ROM (°)				
	Flexion	138.8 (29.6)	124.8 (36.1)	0.144
	Extension	36.0 (17.4)	32.9 (16.1)	0.508
	Abduction	121.0 (31.9)	101.0 (30.6)	0.028
	Adduction	44.0 (22.5)	38.1 (19.1)	0.323
	Internal rotation	53.8 (20.5)	50.4 (23.4)	0.592
	External rotation	45.0 (18.4)	40.4 (21.0)	0.415
EQ-5D-5L score		0.6 (0.2)	0.6 (0.1)	0.715
PCS score		41.2 (6.2)	43.1 (6.2)	0.269
MCS score		42.0 (12.4)	42.1 (10.6)	0.98

[a]The p-values were calculated using the chi-square or Fisher's exact tests.
PPT: Pharmacopuncture therapy, PT: Physical therapy, ROM: Range of motion, EQ-5D-5L: EuroQol 5-Dimension 5-Level, PCS: Physical component summary, MCS: Mental component summary, SPADI: Shoulder Pain and Disability Index, NRS: Numeric rating scale, VAS: Visual analogue scale, BMI: Body mass index.

Table S2. Cost calculation by group

	PPT group (n = 24)		PT group (n = 26)	
	Volume	Cost ± SD	Volume	Cost ± SD
Intervention (24/26)	24 (100)	178 ± 36	26 (100)	92 ± 86
Diagnosis	24 (100)	110 ± 21	26 (100)	134 ± 10
Radiography	24 (100)	8 ± 0	26 (100)	8 ± 0
Compliance	11.2 ± 2.3		11.7 ± 0.9	
Transportation cost		34 ± 24		28 ± 6
Time cost,Total (23/26)	1,249.6 ± 752.6	367 ± 258	1,338.7 ± 565.9	416 ± 190
Travel	857.1 ± 575.2	252 ± 198	988.5 ± 496.9	308 ± 168
Waiting	144 ± 143.0	43 ± 34	66 ± 64.4	19 ± 14
Consultation and treatment	248.5 ± 176.8	74 ± 62	284.2 ± 68.8	89 ± 27
Additional private healthcare usage				
Weeks 1–9 (23/26)	0 (0.0)		1 (3.8)	51 ± NA
Weeks 9–13 (22/26)	3 (13.6)	43 ± 37	1 (3.8)	71 ± NA
Total (23/26)	3 (13.0)	43 ± 37	2 (7.7)	61 ± 14

PPT: Pharmacopuncture therapy, PT: Physical therapy, SD: Standard deviation, NA: Not applicable

Table S3. Overall work impairment according to the Work Productivity and Activity Impairment score

	Week 7	Week 13
PPT group	17.86 (10.74–24.98)	22.81 (14.73–30.90)
PT group	39.99 (33.57–46.41)	39.62 (32.70–46.53)
Difference	22.13 (12.54–31.72)	16.80 (6.17–27.44)
p-value	<0.001	0.003

PPT: Pharmacopuncture therapy, PT: Physical therapy

Table S4. Detailed Treatment of Pharmacopuncture and Phisiotherapy

Intervention type	n (%)	Number of sessions per patient		Dose or duration per session	
		Mean ± SD	Median [IQR]	Mean ± SD	Median [IQR]
**Pharmacopuncture**					
Total	24 (100.0)	11.2 ± 2.3	12.0 [11.0,12.0]	3.2 ± 0.9	3.0 [2.0,4.0]
Shinbaro2	24 (100.0)	8.2 ± 4.1	10.5 [4.0,12.0]	2.5 ± 0.9	2.0 [2.0,3.0]
Jungtong Shinbaro 3	13 (54.2)	8.8 ± 3.6	10.0 [6.0,12.0]	2.1 ± 0.4	2.0 [2.0,2.0]
Hominis Placenta	7 (29.2)	6.7 ± 2.6	8.0 [5.0, 8.5]	2.3 ± 1.0	2.0 [2.0,2.5]
Hwangryunghaedok-tang	3 (12.5)	3.0 ± 3.5	1.0 [1.0, 4.0]	2.0 ± 0.0	2.0 [2.0,2.0]
Shinbaro	1 (4.2)	1.0 ± NA	1.0 [1.0, 1.0]	3.0 ± NA	3.0 [3.0,3.0]
Jungseong Eohyeol	1 (4.2)	1.0 ± NA	1.0 [1.0, 1.0]	2.0 ± NA	2.0 [2.0,2.0]
**Pharmacopuncture in combination**					
Shinbaro2	14 (58.3)	9.5 ± 4.0	11.0 [10.2,12.0]		
Shinbaro2, Jungtong Shinbaro3	10 (41.7)	6.1 ± 3.8	4.5 [3.2, 9.0]		
Hominis Placental, Jungtong Shinbaro3	5 (20.8)	6.2 ± 2.5	7.0 [6.0, 8.0]		
Hominis Placenta	4 (16.7)	3.0 ± 2.2	2.5 [1.8, 3.8]		
Jungtong Shinbaro3	3 (12.5)	6.0 ± 2.0	6.0 [5.0, 7.0]		
Shinbaro2, Hominis Placenta, Jungtong Shinbaro3	2 (8.3)	2.0 ± 1.4	2.0 [1.5, 2.5]		
Hwangryunhaedok-tang	2 (8.3)	4.0 ± 4.2	4.0 [2.5, 5.5]		
Shinbaro	1 (4.2)	1.0 ± NA	1.0 [1.0, 1.0]		
Jungseong Eohyeol	1 (4.2)	1.0 ± NA	1.0 [1.0, 1.0]		
Jungtong Shinbaro3, Hwangryunhaedok-tang	1 (4.2)	1.0 ± NA	1.0 [1.0, 1.0]		
**Acupoints**					
Jianjing (GB21)	21 (87.5)	11.0 ± 2.5	12.0 [11.0,12.0]		
Jianyu (LI15)	21 (87.5)	10.4 ± 3.2	12.0 [11.0,12.0]		
Zhongfu (LU1)	10 (41.7)	10.7 ± 3.4	12.0 [11.2,12.0]		
Jinaliao (SJ14)	7 (29.2)	11.4 ± 0.8	12.0 [11.0,12.0]		
Naoshu (SI10)	6 (25.0)	11.2 ± 2.0	12.0 [12.0,12.0]		
Tianzong (SI11)	6 (25.0)	11.2 ± 0.8	11.0 [11.0,11.8]		
Jianwaishu (SI14)	5 (20.8)	11.2 ± 0.8	11.0 [11.0,12.0]		
Bingfeng (SI12)	5 (20.8)	11.2 ± 0.8	11.0 [11.0,12.0]		
Yunmen (LU2)	1 (4.2)	5.0 ± NA	5.0 [5.0, 5.0]		
**Physical therapy**					
Total	26 (100.0)	11.7 ± 0.9	12.0 [12.0,12.0]	26.0 ± 9.6	29.0 [18.0,30.0]
ICT	22 (84.6)	11.8 ± 0.9	12.0 [12.0,12.0]	13.6 ± 2.2	15.0 [10.0,15.0]
Deep heat therapy	22 (84.6)	11.8 ± 0.9	12.0 [12.0,12.0]	7.0 ± 4.9	5.0 [4.0,15.0]

Laser therapy	11 (42.3)	9.0 ± 1.5	9.0 [8.5,10.0]	11.9 ± 2.4	10.0 [10.0,15.0]
HP	4 (15.4)	11.5 ± 1.0	12.0 [11.5,12.0]	15.0 ± 0.0	15.0 [15.0,15.0]
TENS	4 (15.4)	11.5 ± 1.0	12.0 [11.5,12.0]	15.0 ± 0.0	15.0 [15.0,15.0]
ESWT	1 (3.8)	7.0 ± NA	7.0 [7.0, 7.0]	5.0 ± 0.0	5.0 [5.0, 5.0]
**Physical therapy in combination**					
ICT, Deep heat therapy	22 (84.6)	7.3 ± 4.7	7.0 [3.0,12.0]		
ICT, Laser therapy, Deep heat therapy	11 (42.3)	9.0 ± 1.5	9.0 [8.5,10.0]		
HP, TENS	4 (15.4)	9.8 ± 3.3	11.0 [8.8,12.0]		
ESWT, HP, TENS	1 (3.8)	7.0 ± NA	7.0 [7.0, 7.0]		

ESWT, Extracorporeal; HP, Hot Pack; ICT, Interferential Current Therapy; IQR, Interquartile range; SD, Standard deviation; Shock Wave Therapy; TENS, Transcutaneous Electrical Nerve Stimulation;
